# New York Letter

**Published:** 1892-05

**Authors:** W. L. Hood

**Affiliations:** New York; Nicholson, Ga.


### Correspondence.

### NEW YORK LETTER.

NEW YORK, APRIL 19, 1892.

At last night's meeting of the Section of Practice of the New York Academy of Medicine, Dr. Wilcox of this city read a paper entitled "Anæmia and its Treatment by a New Preparation of Iron." This new preparation is known as the syrup of iron chloride according to the formula of G. W Weld, M. D., of New York. The virtues that Dr. Wilcox claimed for this preparation were that, though sufficiently acid to hold the iron salts in solution, it is not acid enough to destroy the enamel of the teeth. It is composed of five ingredients, viz.: iron, saccharine matter, alcohol, the oil of gaultheria and an alkali which is used to counteract the free hydrochloric acid. He related a series of cases of anæmia that had been treated by this preparation and states that the patients had all been cured in a shorter time than by any other preparation of iron he had ever used. One case was that of a young woman, nineteen years of age, who had suffered from anæmia with shortness of breath on exertion, marked palpitation of the heart, coldness of the hand and feet, and headache. She was given three drams daily of Weld's Iron Preparation. Three weeks later there was no shortness of breath, no palpitation, headache disappeared, and appetite much improved. The hæmoglobin had increased from 46 to 62. Weld's Iron was then increased to half an ounce three times a day and ten days later the hæmoglobin rose to 98 per cent. At the end of this time the patient was discharged cured.

In all the cases treated by him exercise was insisted upon, regular hours for sleep and the functions of the bowels regulated by cascara sagrada and glycerine. From these cases the author of the paper came to the conclusion that in anæmia iron is the best remedy and the tincture of the chloride is the most valuable form. The disadvantages of this preparation such as injury to the teeth, are entirely obviated by the use of Weld's Syrup of the Chloride of Iron, and the especial advantages of the iron are not in any way impaired.

Dr. Weld, the discoverer of this preparation, who was present, said that the syrup of the chloride of iron is not a syrup in the sense of the other syrups that are calculated to upset the stomach and destroy the appetite. Some six or eight years ago while experimenting with the tincture of the chloride of iron, through curiosity he threw a tooth into a solution of the pure tincture and hearing a great deal about the destructive energy of the tincture of iron on the enamel, he took the tooth out after a couple of hours, and the enamel of the tooth was not disturbed in the slightest degree. To make the observation complete, he kept the tooth in the same solution for twenty-four hours and on taking it out he found the enamel in the same condition as before. Then he had his attention called to an old experiment of putting a piece of zinc in strong sulphuric acid, the zinc is not disturbed, but the moment you add water to the sulphuric acid you dissolve the sulphate of zinc and you get immediate chemical action. He then took a dram of the tincture of iron and eight drams of water and immersed the same tooth. The enamel was destroyed in five minutes. From this experiment he was led to the discovery of this new preparation of iron.

Dr. C. E. Quimby said that some few months ago a gentleman who had been under his care called his attention to a preparation called aquzon which was said to be a solution of ozone in water. He took it to a chemist to have it tested for ozone and on being analysed was found to contain about three per cent. per volume of ozone. He, Dr. Quimby, has tried iron in various forms and has been unable to give his patients any amount of the preparation. In combination with this solution of ozone he has been able to raise the quantity of Bland's pills up to twelve per day with very marked benefit. He has used this preparation of ozone in cases of phthisis and was convinced that it acts as ozone in the system. He would try this preparation of iron with ozone, and report the result at a future meeting. Ozone alone, he has found to act upon the system and produce an increase of the hæmoglobin.

Dr. Gottheil, lecturer on dermatology at the New York Polyclinic, uses in all acute inflammatory skin diseases the following mixture with marked success, where pain, burning and intense itching are very prominent symptoms.

℞ Tr. Opii ʒ ss—ʒ 1-8

Oil of Cade ʒ ss.

Carbolic acid gtt. xx.

Simple Syrup ℥ ss.

M. et S.

In chronic noninfections, inflammations such as parenchymatous dermatitis with ulcer, he has found europhen to give very good results as a stimulant to granulating tissue, used in the strength of from one-half a dram to a dram of the powder to an ounce of ointment. In epithelioma as a routine treatment he uses Marsden's paste which is composed of equal parts of arsenious acid and powdered gum-acacia with just enough water to make a stiff paste. A thorough application of this will, he states, cure every case of epithelioma.

Dr. E. L. Keyes has invented a devise which has proved very efficient in his hands in controlling the hemorrhage following the operation of prostatectomy. It consists of a pad made of bichloride gauze. There are four outer layers of gauze six inches square, and six inner layers four inches square. These layers of gauze are fastened together through the centre by a strong silk thread, one end of which is attached to a small white shirt button, and the other end is drawn through the urethra by the aid of a catheter and into the cavity, left by the removal of the prostate. Another silk thread should be attached to the pad by which it can be drawn out through the suprapubic opening after the hemorrhage has been controlled.

Dr. Weir read a paper at the Surgical Section of the New York Academy of Medicine, under the title of "A Unique Derangement of the Knee Joint, Demanding Surgical Interference." The patient was a woman, 45 years of age, who had been under treatment for subacute inflammation of the knee joint, with effusion. She had met with an accident some years ago while out driving when she was thrown forward and struck her knee against the dash-board of the wagon. This accident gave rise to no permanent effects at the time, the resulting synovitis yielding promptly to treatment. Some time later, she reappeared and complained of a tight band about the knee, with a grating sensation. She was unable to lift her foot freely and easily tripped over objects. Examination of the joint showed that the patella when moved seemed to slip over some hard body. An exploratory incision was then resorted to. The synovial membrane was exposed and divided and the opening into the joint enlarged. A satisfactory exploration with the finger could now be made and by raising the patella its inferior surface could be plainly seen. From that surface at the junction of the upper and middle third, a white duplicature was observed hanging down while the bone was thus raised and which rested between the patella and the articular surface of the femur. This thickened fold of membrane was about one-quarter of an inch broad, one-eighth of an inch thick and an inch long. The entire mass was removed, a drainage tube inserted, the synovial membrane sewn up, and the wound closed. The patient regained full power of her limb as a result of this procedure.

---

NICHOLSON, GA., March 5, 1892.

I am much pleased with the sample of Febricide Pills you sent me. I had a case of remittent malarial fever, in which all remedies failed to relieve the pain in the head, and I had made several visits and administered antifebrin, quinine and bromide of potassium, chloral hydrates and morphine, but the pains continued to be very severe, and on the morning that I received your sample of Febricide Pills, I was sent for in haste, the family thought that the patient was taking menengitis, and on my arrival I gave two Febricide Pills, and in fifteen minutes the patient was entirely relieved of all pain. I continued the Pills until the sample was all gone, and the pain never returned, and the patient is now well and gone to business. I never expect to be without them in my practice in the future.

W. L. HOOD, M. D.

